# Inflammatory Mediators in Induced Sputum and Airway Hyperresponsiveness in Cough Variant Asthma during Long-Term Inhaled Corticosteroid Treatment

**DOI:** 10.1155/2012/403868

**Published:** 2012-08-08

**Authors:** Meixuan Liu, Kaixiong Liu, Ning Zhu, Jingwen Xia, Xiaodong Chen

**Affiliations:** ^1^Department of Respiratory Disease, Huashan Hospital, Fudan University, Shanghai 200040, China; ^2^Department of Respiratory Disease, The First Affiliated Hospital, Fujian Medical University, Fuzhou 350005, China

## Abstract

*Objective*. This study aimed to investigate improvements in inflammatory mediator levels in induced sputum and airway hyperresponsiveness (AHR) in cough variant asthma (CVA) during long-term inhaled corticosteroid (ICS) treatment. *Patients and Methods*. Patients with CVA (*N* = 35) and classic asthma (*N* = 26) and healthy subjects (*N* = 24) were recruited into this study. All patients were treated with budesonide (400 **μ**g/day). Measurement of inflammatory mediators in induced sputum and PD20-FEV_1_ (the accumulated provocative dose resulting in a 20% decrease in FEV_1_) in histamine-challenged subjects was performed every three months after the start of medication. Interleukin- (IL-) 5 and IL-10 were assayed by ELISA, and the percentage of eosinophils was detected with Giemsa stain. Trends during the follow-up period were analyzed using a general linear model. *Results*. Inflammatory mediator levels in induced sputum and PD20-FEV_1_ in patients with CVA and classic asthma differed from those in the control group, although no differences were found in the two asthmatic groups. PD20-FEV_1_ significantly increased in CVA patients after ICS treatment for 3 months, while classic asthma patients exhibited a delayed change in AHR. After ICS treatment, levels of IL-5 and IL-10 as well as the percentage of eosinophils in the CVA group were altered at 3 months and 6 months, respectively. Accordingly, the level of inflammatory mediators in classic asthma changed more slowly. *Conclusion*. CVA has a greater improvement in airway inflammation and airway hyperresponsiveness (AHR) than classic asthma with respect to inhaled corticosteroid (ICS). Short-term ICS considerably reduces AHR although longer treatment is required for complete control of airway inflammation.

## 1. Introduction 

Cough variant asthma (CVA), a variant form of classic asthma that presents solely with a cough and responds to bronchodilator treatment, is considered to be the most common cause of chronic coughs [1]. Several studies have suggested that the fundamental pathophysiological characteristics of CVA, airway inflammation and airway hyperresponsiveness (AHR), are identical to those of classic asthma [2]. Furthermore, as a precursor of classic asthma, it has been demonstrated that nearly 30% of untreated CVA patients eventually develop classic asthma. Inhaled corticosteroids (ICSs) are considered to be the first-line therapeutic approach to CVA, which is similar to classic asthma [3]. Previous studies have shown that an inhaled steroid can lead to improvements in markers of airway inflammation as well as in forced expiratory volume in 1 second (FEV_1_) and PC_20_ in mild asthmatics. Budesonide can also reduce the percentage of eosinophils in induced sputum in CVA patients. Furthermore, long-term ICS may prevent the development of classic asthma from CVA by reducing bronchial responsiveness [4]. It is well known that asthma is defined as a complex and chronic inflammatory disorder that is characterized by activation of T helper type-2 (Th2) T cells, IgE production, and eosinophilia [5]. Interleukin- (IL-) 5 is the predominant cytokine associated with eosinophilic inflammation and is of key importance in the pathogenesis of asthma [6]. More recent studies have demonstrated that atopic/asthmatic individuals also have an inherent defect in regulatory T cells, which suppress potentially harmful immune responses by releasing anti-inflammatory cytokines such as IL-10 and TGF-*β* [7]. 

Nevertheless, the changes associated with the improvements in AHR and airway inflammation including regulatory markers in CVA responding to ICS treatment, particularly as compared with those in mild classic asthma, have not yet been elucidated. Moreover, the time course of these changes during treatment is not known. To our knowledge, this is the first study investigating the changes in cytokine levels and hyperresponsiveness during ICS treatment in CVA patients compared with classic asthma patients. In the present study, the levels of Th2-associated cytokines, IL-5 and “cytokine synthesis inhibitory factor” (IL-10), were analyzed. Eosinophils (EOS) in sputum and airway hyperresponsiveness were also determined in CVA patients during the period of ICS treatment. Changes in inflammatory markers and PD20-FEV_1_ (the accumulated provocative dose resulting in a 20% decrease in FEV_1_) in response to histamine challenge were analyzed to evaluate the efficacy of ICS treatment over time in CVA patients compared with mild asthmatics. 

## 2. Materials and Methods

### 2.1. Subjects

Thirty-five patients with CVA and 26 patients with mild classic asthma who were attending the respiratory medicine outpatient clinic of the Huashan Hospital were recruited into this study from November 2010 to May 2011. Twenty-four healthy subjects were simultaneously recruited as the control group. Mild asthma was diagnosed according to the Global Initiative for Asthma (GINA) guidelines [8] and CVA was diagnosed according to the recommendations of the Japanese Cough Research Society [9]. None of the patients had received oral or inhaled steroids previously. Smokers, pregnant women, and individuals diagnosed with upper respiratory tract infections during the preceding 2 weeks were excluded. A separate group of healthy volunteers (14 men and 10 women) attending physical examination department of Huashan Hospital served as healthy controls, all of whom had no past history of asthma or other respiratory diseases and had no current respiratory symptoms. The study protocol was approved by the Ethics Committee of Huashan Hospital, and written informed consent was obtained from all patients and control subjects.

### 2.2. Study Protocol

Prior to the study, all subjects were required to undergo pulmonary function tests, bronchial challenge to histamine, and examination of induced sputum. After participating in these tests, all patients were treated with a daily dose equivalent of 400 *μ*g budesonide. The total follow-up period lasted for twelve months. Levels of IL-5 and IL-10, the percentage of EOS in induced sputum, and the value of PD20 were examined every 3 months after the medication. This study comprised a longitudinal comparison of the changes in airway inflammation and bronchial hyperresponsiveness in CVA and classic asthma patients to estimate the effect of ICS treatment.

### 2.3. Pulmonary Function and Histamine Challenge Tests

FEV_1_ and forced vital capacity (FVC) were measured utilizing a computerized spirometer (Master Screen Body, Jaeger, Germany) according to the standardization criteria of the American Thoracic Society [10]. To be eligible for the study, forced expiratory volume in 1 s (FEV_1_) was required to be 80% or more of the predicted value.

Bronchial responsiveness was tested using an aerodynamic particle sizer (APS) aerosol provocative system (Master Screen Body, Jaeger, Germany). PD20-FEV_1_ was measured by inhalation histamine challenge with different concentrations of histamine (0.03–7.8 mg/mL) delivered by the quantitative aerosol dosimeter of the APS system. Initially, baseline FEV_1_ was measured using a spirometer 2 minutes after inhalation of a control solution (0.9% saline). Subjects inhaled a histamine aerosol from a nebulizer with tidal breathing whilst wearing a nose clip for 2 min. Total inhalations of each concentration were administered, and FEV_1_ was measured three minutes after each period of inhalation. The test was stopped in the event of a fall in baseline FEV_1_ of 20% compared with the control inhalation solution. PD20 was calculated as the provocative dose of histamine causing a 20% fall in FEV_1_. AHR was considered to be present in subjects with PD20 values less than 2.2 mg. Subjects received two puffs (200 *μ*g) of salbutamol from a metered dose inhaler following the histamine challenge test.

### 2.4. Sputum Induction and Processing

Sputum was induced with an aerosol of hypertonic saline solution according to a previously described protocol [11]. Collected sputum samples were processed within 2 hours. The volume of mucus plugs was measured prior to incubation with four volumes of 0.1% dithiothreitol (DTT). Samples were then vortexed gently and incubated in a shaking water bath at 37°C for 15 min to ensure complete homogenization. The resulting suspensions were filtered through nylon gauze (48 *μ*m), and total leukocyte cell counts and cell viability were determined. Filtrates were centrifuged at 750 ×g for 10 min, and supernatants were aspirated and stored in eppendorf tubes at −80°C for IL-5 and IL-10 assays. Cell suspensions were adjusted to 1.0 × 10^6^/mL and a sample (60 *μ*L) was used for cytocentrifuge preparations. Slides were stained with Giemsa stain, and 400 differential nonsquamous cell counts were performed. Results were expressed as the percentage of eosinophils in the total nonsquamous cell count. Samples containing more than 30% squamous cells were excluded from the analysis.

### 2.5. Measurement of IL-5 and IL-10

Concentrations of IL-5 and IL-10 in sputum supernatants were determined using a solid-phase sandwich enzyme-linked immunosorbent assay (ELISA) (PeproTech, NJ, USA). The sensitivities of the assays as quoted by the manufacturer were 15 pg/mL and 10 pg/mL, respectively. 

### 2.6. Statistical Analysis

Statistical analyses were performed using SPSS 17.0 for Windows. All data were expressed as the mean ± standard deviation (SD) or median (M) with the range. A general linear model was applied for comparison of the time course of sputum marker levels and PD20 before and after treatment in patients with CVA and asthma. A time course of repeated measurements was used to compare groups. Statistical significance identified between groups or time points by post hoc analysis was further analyzed by Bonferroni's tests. *P* < 0.05 was considered statistically significant.

## 3. Results 

### 3.1. Clinical Characteristics

In the current study, 28 patients with CVA, 23 classic asthmatics, and 22 healthy subjects successfully completed the sputum induction procedure and follow-up period. Clinical features of the three study groups are shown in Table 1. Higher serum total immunoglobulin E (IgE) levels and blood eosinophil counts were detected in the two asthma groups compared with the control group. There were no significant differences between the CVA and asthma groups with respect to age, sex, total IgE, FEV_1_% predicted, FEV_1_/FVC, hemocytes, and atopy (Table 1). 

### 3.2. Bronchial Provocative Tests

**Figure 1:**
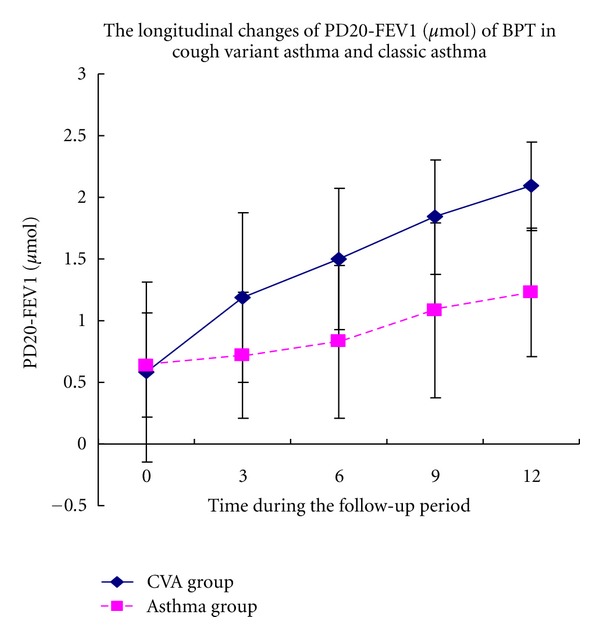
Longitudinal changes in inflammatory mediators in induced sputum during the follow-up period in the CVA and classic asthma groups.

Initially, no significant differences were identified between patients with CVA and classic asthma in terms of PD20-FEV_1_ in BPT. PD20-FEV_1_ significantly increased from 0.58 ± 0.73 *μ*mol to 1.19 ± 0.69 *μ*mol in CVA patients after receiving ICS for 3 months. However, in asthmatic patients PD20-FEV_1_ was changed after treatment for 9 months. ICS treatment for 12 months significantly improved airway hyperresponsiveness in patients with CVA and classic asthma although the overall level of PD20-FEV_1_ in CVA patients was higher than that in classic asthma patients after treatment (Figure 1).

**Figure 2:**
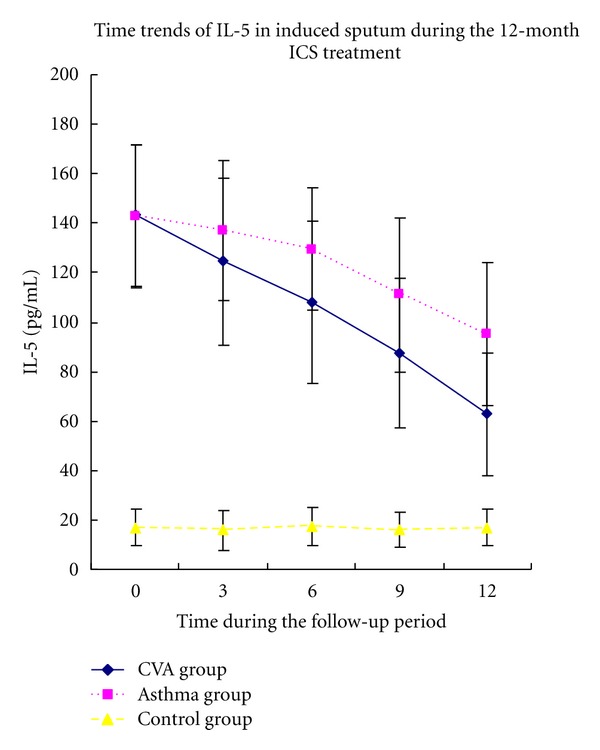
Time course of IL-5 in induced sputum followed for 12 months of ICS treatment in CVA and asthma groups.

**Figure 3:**
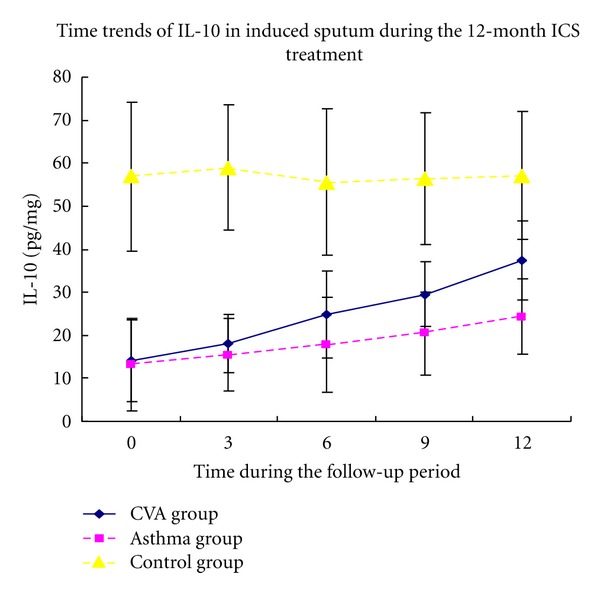
Time course of IL-10 in induced sputum followed for 12 months of ICS treatment.

Compared with the control group, the CVA and classic asthma groups were found to have higher baseline levels of IL-5 and percentage of EOS in addition to lower concentrations of IL-10 in induced sputum(Figures 2 and 3). No significant differences were observed between the two asthma groups.

Levels of inflammatory markers were improved to some extent in the two asthmatic groups following treatment with budesonide. However, the changes in inflammatory mediators in induced sputum differed between the two groups during the period of medication. At each time point, levels of inflammatory markers in the CVA group also differed from those in the classic asthma group (*P* < 0.05).

The overall level of IL-5 in the CVA group was lower than that in the classic asthma group. Besides, the IL-5 levels in the CVA group decreased after treatment for 3 months while those in classic asthma group began to decrease after treatment for 6 months. Higher levels of IL-10 were detected in the CVA group compared with those observed in the classic asthma group throughout the observation period. IL-10 values significantly increased in the CVA group after ICS treatment for 6 months, whereas levels in the classic asthma group increased after treatment for 12 months. 

**Figure 4:**
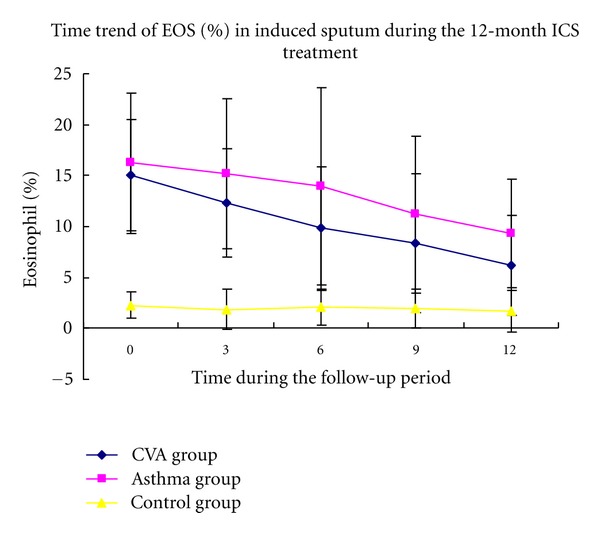
Time course of EOS (%) in induced sputum followed for 12 months of ICS treatment.

The percentage of eosinophils (EOS%) in sputum was lower in the CVA group compared with that in the classic asthma group after treatment. The EOS% significantly decreased after ICS treatment for 6 months in the CVA group, while a change was observed in the classic asthma group only after treatment for 12 months (Figures 2, 3, and 4).

## 4. Discussion

Cough variant asthma is essentially an occult phase of classic asthma that shares similarity in fundamental pathologic and pathophysiologic characteristics. Evidence suggests that an imbalance in Th1 and Th2 lymphocyte responses plays a critical role in the pathogenesis of chronic asthma [12]. The potential of Th2 cells to promote allergic immunopathology is amplified by production of a range of mediators, which together promote the salient features of asthma such as IgE production, AHR, inflammation, and tissue remodeling [13]. In addition to the traditionally accepted Th2 immune response mechanism of asthma, recent advances in both immunology and clinical phenotyping of asthma have raised the possibility that other mechanisms may drive pathology in some patients or coexist with Th2-type inflammation. There is now evidence for a variety of suppressive or regulatory T cell (Treg) subsets that suppress potentially harmful immune responses through the release of various anti-inflammatory cytokines including IL-10 and TGF-*β* [7, 14, 15]. However, the effect of regulatory T cells has not been specifically detected in patients with CVA. Therefore, on the basis of detailed prior studies of the immunopathogenesis of asthma, we hypothesized that CVA exhibits homogeneity with classic asthma. The current study was conducted to further assess inflammation in CVA by analysis of induced sputum levels of anti-inflammatory Th2-cytokines (IL-5 and IL-10) in addition to measurement of the numbers of eosinophils as the immune effector cells in this condition. Furthermore, changes in the levels of these inflammatory mediators during the period of ICS medication were investigated. It is believed that ICS forms the basis of maintenance therapy for patients with CVA [16]. The effect of ICS on CVA in terms of airway hyperresponsiveness and sputum inflammatory markers was investigated in contrast to those in classic asthma. 

In this study, the basic level of IL-5 and percentage of eosinophils in CVA patients and mild asthmatics were clearly higher than those in the control group although no statistically significant differences were detected between the two asthmatic groups. IL-5, one of the most important Th2-associated cytokines, is critical for the survival, differentiation, proliferation, and maturation of eosinophils within the bone marrow. Activated eosinophils possess secondary granules containing four primary cationic proteins: major basic protein (MBP), eosinophil cationic protein (ECP), eosinophil-derived neurotoxin (EDN), and eosinophil peroxidase (EPO), all of which participate in the processes of airway inflammation [17]. Increased eosinophil counts in sputum have been reported by Niimi et al. [18]. Therefore, our results are consistent with previous studies indicating that eosinophilic inflammation is involved both in CVA and in classic asthma. It is remarkable that not all asthma can be explained by the predominant Th2 and eosinophil-dominant inflammation [19]. Accumulating evidence has suggested that Th17 cells and Th17 cytokines are involved in the regulation of asthmatic inflammation as well [20, 21]. Th17 is a third subset of CD4^+^ T-helper lymphocytes characterized by the production of the IL-17 family cytokines like IL-17A and IL-17F. Animal models of asthma denote that Th17 cells may promote Th2 immunity and exacerbate airway hyperresponsiveness [22, 23]. Additionally, sputum neutrophils were the highest in moderate-severe asthmatics compared with mild asthmatics and IL-17A necessarily led to neutrophilic inflammation in severe asthma [24]. Taken together, these findings showed that Th17 plays a key role in asthma pathogenesis, particularly in the severe form of asthma. In the present study, we observed elevated sputum IL-5 and eosinophils (%) in CVA and mild asthmatics, perhaps merely supporting that eosinophilic inflammation is involved dominantly in the mild phase of the disease. We speculate that the role of Th2 and Th17 cells might correlate with the severity of asthma. Moreover, there are also different inflammatory subtypes in asthma according to the assessment of induced sputum [25]. Apart from eosinophils independent impact, Matsuoka et al. [26] reported that eosinophil could act in synergy with neutrophils as well. They also found that inflammatory subtypes in CVA were associated with the maintenance ICS doses. Eosinophilic subtypes required higher maintainenance dose of ICS but represented a short-term preferable response of treatment with ICS than noneosinophilic asthma [27]. In the present study, we did not assess inflammatory cells like neutrophils, just aiming to principally elucidate the response of eosinophilic inflammation with respect to long-term ICS treatment. A good yet dissimilar improvement was found in eosinophilic inflammation under 12-month ICS treatment between CVA and classic asthma, providing some insight into the efficacy of long-term ICS on eosinophilic inflammation in CVA.

We also observed a kind of immune modulator, the level of induced sputum IL-10 in the three studied groups. IL-10, an anti-inflammatory cytokine secreted predominantly by immune regulatory cells in the lung, inhibits the transcription of many proinflammatory cytokines and chemokines [28] Borish et al. [29] reported that IL-10 secretion by alveolar macrophages may be impaired in asthmatic patients. In our study, lower concentrations of IL-10 were observed in CVA patients and mild asthmatics compared with control subjects, suggesting the existence of deficient immune regulation in cough variant asthma as well as classic asthma. These data also indicate that ICS treatment for 12 months in CVA patients significantly altered the levels of induced sputum IL-5 and IL-10 and reduced the number of eosinophils. Despite the similarity in the pattern of inflammatory sputum markers in patients with asthma and CVA, the disparity of the two groups is yet detected in terms of improvement of the same item under glucocorticoid treatment. The present study clearly demonstrated greater amelioration of airway inflammation in CVA compared with mild classic asthma, by the apparent differences in levels of the markers investigated over the period of ICS medication. These data indicate that airway inflammation in CVA is highly responsive to early ICS treatment and that may also be true for cases of mild classic asthma.

In this study, the improvement of airway hyperresponsiveness in patients with CVA and classic asthma was also investigated. Challenge tests are an important measurement for diagnosis and evaluation of the severity of CVA. Histamine was used as the provocative agent in this study. In accordance with previous reports [30], the PD20-FEV_1_ of CVA significantly increased after budesonide treatment for only 3 months, and, furthermore, therapy continued for 12 months resulted in improved airway hyperresponsiveness such that it was detected only to an extremely mild degree. In contrast, the improvement in PD20-FEV_1_ observed in classic asthma was delayed and was only apparent after treatment for 9 months. It is postulated that these differences are due to thickening of the subbasement membrane in CVA patients compared with healthy controls, although the degree of thickening was less than that detected in classic asthma [31]. This may result in reversible tracheole airflow obstruction and mild airway hyperresponsiveness. This study further demonstrated that early and sufficient treatment with ICS effectively reduces AHR and suppresses airway inflammation in CVA patients. Moreover, this accounts for the close association between AHR and chronic airway inflammation with airway remodeling. Treatment with ICS for 3 months may significantly decrease AHR in CVA, although, for most patients, long-term ICS treatment remains indispensable for complete elimination of airway inflammation.

There are certainly other antiasthma mediations such as cysteinyl leukotriene (cys-LT) receptor antagonists (LTRAs). LTRAs alleviate clinical symptoms and improve pulmonary function as well as airway inflammation in classic asthma [32], but montelukast for 4 weeks was yet unable to change pulmonary function, AHR, and sputum mediator levels in CVA other than sputum eosinophil count and cough sensitivity [33]. But for the long-term outcome according to our study, we were satisfied to notice that ICS for 12-month monotherapy predominantly attenuates AHR, sputum mediator levels as well as sputum eosinophil count both in CVA and classic asthma. Thereof our study supported the concept that ICS is superior to montelukast with respect to sputum eosinophil and hyperresponsiveness.

Nevertheless, some limitations of our study should be noted. In the present study, we lack determining the changing trend of cough sensitivity in CVA under long-term ICS treatment. It might have affected the precise evaluation of the efficacy of ICS treatment. Actually, there is evidence that cough threshold did not change whether taking or not taking ICS [4]. Thus we may draw a preliminary conclusion that, compared with LTRAs, inhaled corticosteroids play a better role in AHR and airway inflammation rather than cough receptor sensitivity in CVA. The intriguing comparison of budesonide with montelukast further confirmed that ICS possibly better ameliorates the degree of sputum inflammatory mediators in cough variant asthma. However, the detailed mechanisms remain to be investigated by further studies.

To summarize, ICS treatment provides greater alleviation of Th2-associated allergic responses and repair of regulatory mediators in CVA than in classic asthma. The current study has indicated that airway hyperresponsiveness can be rapidly improved as a result of short-term ICS treatment, although complete control of airway inflammation requires longer-term ICS treatment. Further studies, including randomized placebo-controlled trials, are required to confirm the effective maintenance period and dose of ICS treatment in CVA. 

## Figures and Tables

**Table 1:** Clinical characteristics of the three studied groups.

	CVA group (*N* = 28)	Asthma group (*N* = 23)	Control group (*N* = 22)
Sex (M : F)	28 (13 : 15)	23 (10 : 13)	22 (12 : 10)
Age (years)	42.96 ± 14.23	40.73 ± 11.41	38.76 ± 13.18
Total IgE (IU*·*mL^-1^)	657.85 (24.00 ~ 2342.00)^a^	593.74 (37.40 ~ 2592.00)^a^	65.46 (17.34 ~ 153.00)
FEV_1_% predicted	93.76 ± 12.72	90.24 ± 15.11	102.12 ± 14.49
FEV_1_/FVC	82.67 ± 4.76	84.13 ± 6.92	89.21 ± 4.7
Leukocyte (10^9^/L)	6.65 ± 1.78	7.06 ± 1.91	6.71 ± 1.72
Neutrophil (%)	56.85 ± 9.52	60.72 ± 9.47	57.39 ± 7.20
Eosinophil (%)	7.28 ± 2.46^a^	6.86 ± 2.58^a^	3.08 ± 1.91
Basophil (%)	0.092 ± 0.076	0.074 ± 0.062	0.088 ± 0.049
Monocytes (%)	6.29 ± 1.05	6.47 ± 1.90	6.51 ± 1.35
Lymphocytes (%)	29.55 ± 8.83	33.16 ± 14.27	27.86 ± 15.23
Atopy	12/16^ a^	10/13^a^	0/22

^a^ *P* < 0.05 compared with controls.
